# Uptake of postpartum modern family planning and its associated factors among postpartum women in Ethiopia: A systematic review and meta-analysis

**DOI:** 10.1016/j.heliyon.2021.e08712

**Published:** 2022-01-04

**Authors:** Azimeraw Tesfu, Fentahun Beyene, Fikadu Sendeku, Kihinetu Wudineh, Getnet Azeze

**Affiliations:** a Department of Midwifery, College of Medicine and Health Sciences, Bahir Dar University, Bahir Dar, Ethiopia; b Department of Midwifery, College of Health Sciences, Woldia University, Woldia, Ethiopia

**Keywords:** Contraceptive, Prevalence, Utilization

## Abstract

**Objective:**

This study aimed to estimate the pooled prevalence and factors associated with postpartum modern contraceptive use in Ethiopia.

**Design:**

Systematic Reviews and Meta-Analysis.

**Method:**

PubMed, MEDLINE, EMBASE, Hinari, Google Scholar, direct Google search, African Journal Online (AJOL), an online repository, and gray kinds of literature were used for searching. This meta-analysis included eighteen cross-sectional studies. The quality appraisal criterion of the Joanna Briggs Institute (JBI) was employed to critically appraise papers. The I2 statistics were used to test heterogeneity and subgroup analysis was computed with the evidence of heterogeneity. The Egger test with funnel plot was used to investigate publication bias. The “generate” command in STATA was used to calculate the logarithm and standard error of the odds ratio (OR) for each included study. Then odds ratio (OR) with a 95% confidence interval (CI) was presented.

**Result:**

Eighteen studies were included in the systematic review and meta-analysis. The pooled prevalence of modern postpartum family planning utilization among postnatal women in Ethiopia was 45.44% (95%CI: 31.47, 59.42).

Prenatal family planning counseling (AOR = 3.80; 95%CI: 2.70, 5.34), postnatal care utilization (AOR = 3.07; 95%CI: 1.39, 6.77), spouse communication on family planning (AOR = 1.86; 95%CI:1.36,2.54), resumption of menses (AOR = 4.20; 95%CI: 2.95, 5.99), and resumption of sexual activity (AOR = 3.98; 95%CI: 2.34, 6.79) were associated factors to uptake modern postpartum family planning among postnatal women.

**Conclusion:**

The pooled prevalence of postpartum modern contraceptive use was low. The most common factors significantly associated with postpartum modern contraceptive use were prenatal family planning counseling, postnatal care utilization, spouse communication on family planning, resumption of menses, and resumption of sexual activity were the commonest factors significantly associated with postpartum modern contraceptive use.

## 1 Introduction

Family planning (FP) is an important component of prenatal care, postpartum care, and the first year after delivery. The term "postpartum family planning" (PPFP) refers to the beginning of family planning services within the first 12 months after childbirth in order to avoid closely spaced and unplanned pregnancies [1, 2]. Even when breastfeeding, postpartum women can experience amenorrhea, or the lack of menses, for variable durations of time, and their fertility can recover before menses restart. Pregnancy can happen within 45 days following giving birth for women who are not breastfeeding [3]. When a woman transitions from exclusive nursing to nearly complete breastfeeding, her chances of becoming pregnant improve. Using family planning (FP) during the first year after giving birth has the potential to prevent at least some of these unwanted births significantly [4].

In developing nations, almost 95% of postpartum women wish to postpone pregnancy for at least two years, and 70% of women who are 0–12 months postpartum want to avoid pregnancy in the following 12 months but are not using contraception [5]. Every year, hundreds of thousands of women die as a result of pregnancy and/or childbirth problems around the world. Almost all maternal deaths (99 percent) occur in underdeveloped countries, with Sub-Saharan Africa accounting for more than half (66 percent) of these deaths [6]. Ethiopia has one of the highest maternal mortality ratios (MMR) in Sub-Saharan Africa, with 412 maternal deaths per 100,000 live births [7]. According to a study conducted in 172 countries, the number of maternal deaths would have been 1•8 times higher if contraception had not been used, implying that contraception prevented 44 percent of maternal deaths. If the unmet need for contraception is satisfied, there could be a 29 percent reduction in maternal deaths per year [8]. Pregnancies in the first 12 months after birth are more likely to result in unfavourable health outcomes for the mother and child, such as stillbirth, preterm birth, low birth weight, and small size for gestational age [9], as well as linked to an increased risk of induced abortion [10]. [10]. Shorter birth intervals are also associated with a high risk of malnutrition, and infant mortality [11]. As a result of these substantial health risks, spacing pregnancies by at least two years can save 10% of infant deaths and 21% of mortality in children aged 1–4 years worldwide [9]. As a result, the World Health Organization recommends that pregnancies be spaced by at least 24 months [12]. Furthermore, while postpartum family planning (FP) is internationally recognized as a critical intervention for reducing unmet needs and maternal and child mortality and morbidity, the majority (61%) of postpartum women in low- and middle-income countries have a prospective unmet need; in Ethiopia, this rises to a full 74 % (nearly half (47%) of postpartum women for spacing who have short (<23 months) birth-to-pregnancy intervals and 27 % for limiting) [13].

According to the Demographic and Health Survey (DHS), the prevalence of modern postpartum family planning utilization is 23%, showing that contraceptive service delivery during the postpartum period still has a considerable gap [14]. Therefore, this review aimed to summarize the findings of all relevant individual studies regarding prevalence and factors associated with PPFP utilization in Ethiopia. Therefore, the available evidence obtained from this systematic review and meta-analysis may provide convincing and reliable information on the epidemiology and overall factors that limit PPFP utilization in Ethiopia.

## 2 Methods

### 2.1 Study setting

Ethiopia is one of the east African countries in the horn of Africa, with nine national regional states (Tigray, Afar, Amhara, Oromia, Somalia, Benishangul-Gumuz, Southern Nations Nationalities and People Region (SNNPR), Gambella, and Harari), as well as two administrative states (Addis Ababa City administration and Dire Dawa city administration) [15]. The national regional states, as well as the two cities' administrative councils, are further divided into eight hundred woredas and around 15,000 kebeles. It has a land area of 1.104 million k.m^2^ and a population of more than 108 million people, with more than 80.08 percent of the population live in rural areas [16].

### 2.2 Criteria for eligibility

The systematic review and meta-analysis included

✓ The study looked at postpartum women who used one or more modern contraceptive methods within the first 12 months after giving birth. Articles dealing with any kind of study design (cross-sectional or cohort)
✓ Papers published in the English language and unpublished studies on postpartum women in Ethiopia were conducted before April 30, 2019.
✓ Studies reported if one or more of the following outcomes were reported: PPFP utilization, postpartum modern contraceptive use; the unmet need for FP; additionally, studies that included data on predictors or barriers of contraceptive use were included in the review.
✓ Whereas articles without full abstracts or texts, or articles reported without regard to the outcome of interest, were excluded.
✓ Primary studies satisfied 60% of the Joanna Briggs Institute (JBI) criteria for measuring the quality of primary research in the meta-analysis [17].

### 2.3 The outcome of the measurement

The primary outcome of this study was the pooled prevalence of postpartum modern contraceptive use. **Postpartum modern contraceptive uptake:** When a postpartum mother claimed to have regularly used contemporary contraception method within 12 months [1, 18], such as sterilization (male or female), intrauterine devices, subdermal implants, oral contraceptives, injectables, or condoms (male or female).

The second outcome of interest in this study was factors associated with postpartum modern contraceptive use. The adjusted odds ratio was calculated for the common factors of the reported studies with 95%CI.

### 2.4 Strategy for search

This systematic review and meta-analysis were conducted based on the review of different kinds of literature in Ethiopia. The study selection techniques were outlined using the Preferred Reporting Items for Systematic Reviews and Meta-Analysis (PRISMA) flow diagram to ensure scientific rigor [19].

Studies conducted in Ethiopia from 2012 to 2019 on women with PPFP utilization were searched using the following search strategies: PubMed, MEDLINE, EMBASE, Hinari, Google Scholar, direct Google search, African Journal Online (AJOL), online repository, and gray pieces of literature were used. To restrict the search, the main search phrases were joined using the Boolean (Search) operators "AND" and "OR." The search strategy included the use of title/abstract related to medical subject headings or the key search terms included: postpartum OR post-delivery OR parturition OR puerperium OR prevalence OR magnitude OR proportion OR use OR utilization OR intention OR unmet need OR barrier AND Predictors OR contraception, OR contraceptive, OR family planning, OR modern contraceptives, OR modern postpartum family planning, OR modern family planning, AND Ethiopia and related were searched. Furthermore, We manually searched for gray literature and other relevant data sources, such as unpublished thesis/papers with predicted publication dates.

### 2.5 Data extraction

**Figure 1:**
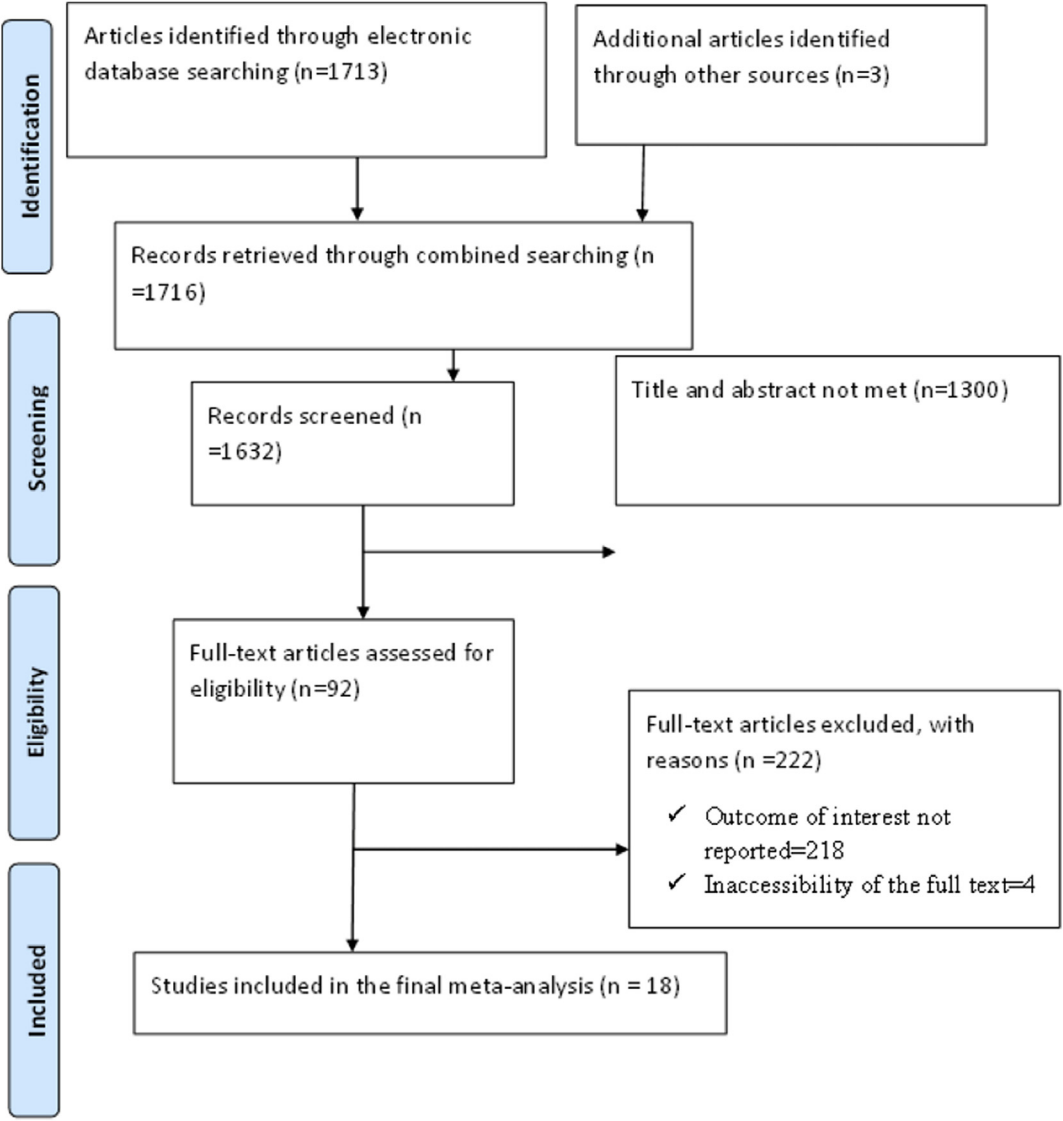
A schematic presentation of the PRISMA flow diagram used to select and include studies in 2019.

Articles were reviewed; relevant information was extracted from identified pieces of literature and summarized using the Preferred Reporting Items for Systematic Reviews and Meta-Analyses (PRISMA) protocol (Figure 1).

Data was retrieved using a Microsoft Excel spreadsheet with a pre-piloted data extraction structure. Three independent authors (AAT, FYB, and FWS) extracted data from the included studies and checked the data together. From each included study, the tool consisted of information on author/s name, year of publication, year of study, study area, study region, study design, sample size, time of point assessment, the prevalence of PPFP utilization, and information regarding the associated factors were extracted. The Joanna Briggs Institute's Critical Appraisal Tools for Review addressing concerns of prevalence in systematic reviews and meta-analysis were used to assess the quality of included studies. Two reviewers (GGA and KGW) independently critically appraised the quality of each article using a critical appraisal checklist adopted from JBI. If any discrepancies during critical appraisal arise, all reviewers have discussed and resolved the discrepancies.

Among the methodological components assessed are addressing the target population, appropriateness of participant recruitment, adequacy of sample size, a detailed description of study subjects, data analysis with sufficient coverage of the identified sample, use of valid methods for condition identification, measurement of the condition in a standard and reliable way for all participants, use of appropriate statistical analysis, and adequacy of response rate [17].

### 2.6 Data analysis

For meta-analysis, the retrieved data were loaded into Microsoft Excel and subsequently into STATA version 11 statistical software.

The impact of a few associated factors was also investigated independently. We used adjusted odds ratio estimates with the confidence interval (CI) as the measure of association for the meta-analysis. The prevalence rates and odds ratio (OR) were used to estimate and assess the overall pooled effect size (i.e. estimations of the prevalence and factors) of PPMC use with a 95 percent confidence interval (CI). The random-effects model of analysis was used to conduct the meta-analysis since it minimizes the heterogeneity of the included studies [20]. The variance of the study-specific prevalence was stabilized with the Freeman-Tukey double arcsine transformation before pooling the data in a random-effects meta-analysis model to limit the effect of studies with extremely small or extremely large prevalence estimates [21]. The presence of publication bias was assessed using a funnel plot and Egger's test, with a p-value of less than 0.05 indicating statistically significant publication bias [22]. Statistically significant heterogeneity was defined as a p-value of less than 0.05 [23,24]. The I2 test results and corresponding p-value were used to assess study heterogeneity [25]. The I2 statistics are used to determine how much of a study's total variation is due to real between-study variations rather than chance. The I2 score ranges from 0 to 100 percent, with an I2 of 75 percent indicating significant heterogeneity among studies [23]. Furthermore, subgroup analysis was used to determine the source of difference among studies by grouping characteristics such as study year, study environment, study design, and mean sample size. Results.

### 2.7 Description of the included studies

A total of 1716 articles were searched using different online databases and gray pieces of literature. From all identified studies, 84 duplicate articles were identified and expunged. After reviewing titles and abstracts, 1300 irrelevant articles were excluded because their titles were not related to the topic of interest, 222 studies were discarded for various reasons. Furthermore, 218 papers were omitted from the meta-analysis due to the lack of a relevant outcome, and four studies were excluded due to the inaccessibility of complete texts. Finally, this systematic review and meta-analysis comprised a total of 18 papers. The investigations were done in Ethiopia's several areas, including one in Ethiopia's Somali region [26], three in the Southern Nations, Nationality and Peoples Region (SNNPR) [27, 28, 29], five in Tigray [30, 31, 32, 33, 34], eight in Amhara [35, 36, 37, 38, 39, 40, 41, 42], and one in Addis Ababa, Ethiopia's capital city [43]. In terms of study design, sixteen studies selected for the analysis were cross-sectional whereas two studies were prospective cohort studies. Also, twelve studies were community-based and six studies were institutional-based (Table 1). The PRISMA Flow Diagram was used to guide the overall selection process of investigations (Figure 1).

**Table 1:** Description of study characteristics included in the systematic review and meta-analysis in different regions of Ethiopia from 2012 to 2019.

S.N	Author with study year	Region	Study design	Sample size	The time point of assessment	Outcomes measurement	Prevalence
1	Niguse et al. (2015) [26]	Somali	CBCS	556	0–12 months	PPFP utilization	12.3
2	Dona et al. (2017) [27]	SNNP	CBCS	695	0–12 months	PPC utilization	31.7
3	Marta B. et al. (2015) [37]	Amhara	INCS	404	0–12 months	PPMC utilization	45.8
4	Abraha TH et al. (2015) [30]	Tigray	CBCS	601	0–12 months	PPMC use	48
5	Gizaw W et al. (2014) [39]	Amhara	CBCS	829	0–12 months	PPMC utilization	46.7
6	Gebremedhin et al. (2015) [43]	AA	CBCS	803	0–12 months	PPMC use	92.80
7	Gejo NG et al. (2018) [18]	SNNP	INCS	368	0–12 months	PPMC use	77.9
8	Demie et al. (2016) [38]	Amhara	INCS	248	6–14 weeks	PPFP utilization	41.6
9	Abera et al. (2013) [40]	*Amhara*	CBCS	703	0–12 months	PPC use	48.4
10	Taye et al. (2018) [36]	Amhara	CBCS	550	6–12 months	PPMC utilization	63
11	Assefa et al. (2019) [34]	Tigray	CBCS	422	0–12 months	PPC utilization	48.6
12	Gurja Embafrash G et al. (2014) [33]	Tigray	INCS	409	0–12 months	Unmet Need, PPMC use	29.3
13	Mengesha et al. (2012) [41]	Amhara	CBCS	899	0–12 months	PPC use	10.3
14	Zimmerman et al. (2017) [29]	SNNP	CB PC	329	0–6 months	PPFP counseling	41.5
15	Tegegn et al. (2014) [42]	Amhara	CBCS	383	0–12 months	Unmet Need, PPMC use	54.7
16	Gebremariam et al. (2015) [31]	Tigray	INCS	605	6–12 months	PPC use	68.1
17	Tafere et al. (2016) [35]	Amhara	IN PC	970	0–6weeks	PPFP use	19.1
18	Abraha et al. (2017) [32]	Tigray	CBCS	1109	0–12 months	PPC use	38.3

NB: CBCS: Community Based Crossectional Study; CBPC: Community Based Prospective Cohort; INCS: Institutional based Crossectional study; INPC: Institutional Prospective Cohort; PPC: Postpartum Contraceptive.

### 2.8 The overall pooled prevalence of postpartum modern contraception (PPMC) use

**Figure 2:**
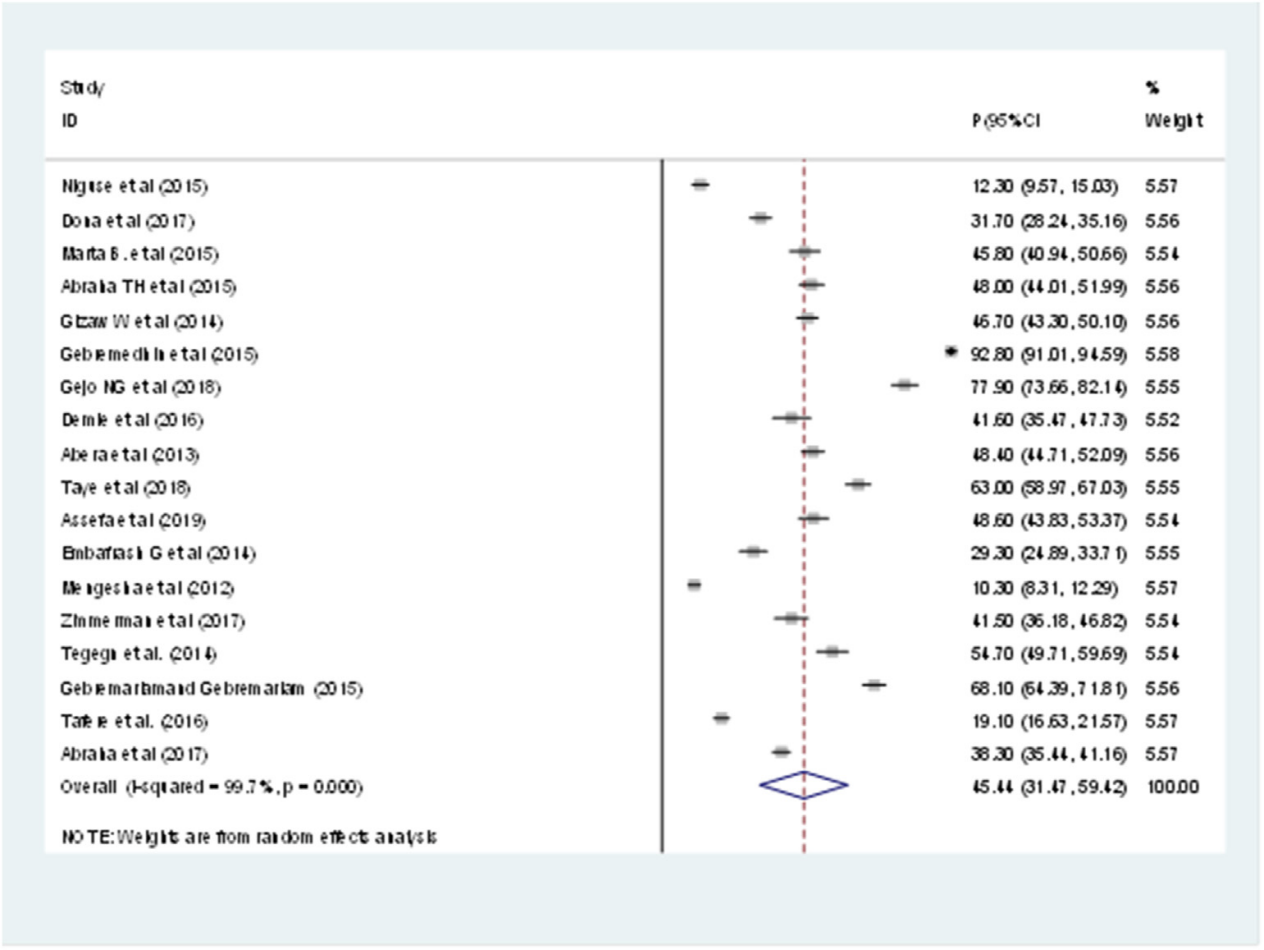
Postpartum modern contraceptive use in Ethiopia, 2019.

The fixed-effect model revealed significant variation in the prevalence of PPMC usage in Ethiopia when the results of 18 researches were pooled. Accordingly, the overall pooled prevalence of PPMC use in Ethiopia was 45.44% (95%CI: 31.47, 59.42) with (I^2^ = 99.7%, p-value ≤0.001) (Figure 2).

### 2.9 Subgroup meta-analysis

We further conducted a subgroup meta-analysis to identify the possible source of this considerable heterogeneity using different study characteristics: study year, study design, study setting, and mean sample size (Table 2).

**Table 2:** Sub-group analysis on the prevalence of PPMC utilization in Ethiopia, 2019 (n = 18).

Variables	Number of included studies	Total sample size	Estimate (95% CI)	Heterogeneity statistics	
				I2	P-value
**By study year**					
<2016	9	6192	45.64 (23.47, 67.81)	99.8	≤0.001
≥2016	9	4691	45.18 (31.43, 58.94)	99.1	≤0.001
**By study design**					≤0.001
Cross-sectional	16	9584	47.34 (32.17, 62.51)	99.7	≤0.001
Prospective Cohort	2	1299	30.17 (8.22, 52.12)	98.2	≤0.001
**By study setting**					≤0.001
Community	13	8484	46.49 (29.11, 63.86)	99.7	≤0.001
Institutional	6	2399	42.72 (20.52, 64.92)	99.3	≤0.001
**By sample size**					≤0.001
≥605	9	7169	40.85 (18.27, 63.44)	99.8	≤0.001
<605	9	3714	50.08 (40.39, 59.77)	97.4	≤0.001

### 2.10 Common modern postpartum family planning in Ethiopia

In this systematic review and meta-analysis; injectable (Depot medroxy progesterone acetate) 48.86% (95%CI:36.42, 61.30, I^2^ = 99.5%, p ≤ 0.001), implants 24.87% (95%CI:18.04, 31.70, I^2^ = 98.7%, p ≤ 0.001), oral contraceptive pills 9.69% (95%CI:6.61, 12.77, I^2^ = 97.9%, p ≤ 0.001), IUCD 8.50% (95%CI:5.52, 11.48,I^2^ = 97.1%, p ≤ 0.001) were the most often modern contraceptive methods utilized by Ethiopia postpartum mothers.

### 2.11 Publication bias

**Figure 3:**
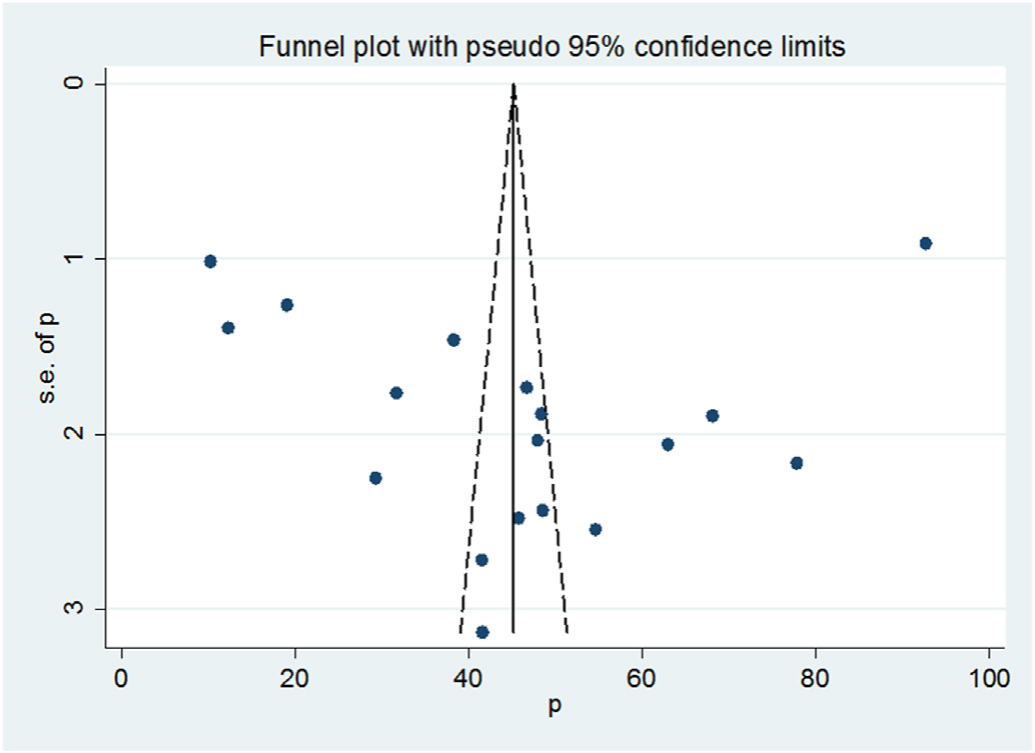
Funnel plot displaying publication bias.

**Figure 4:**
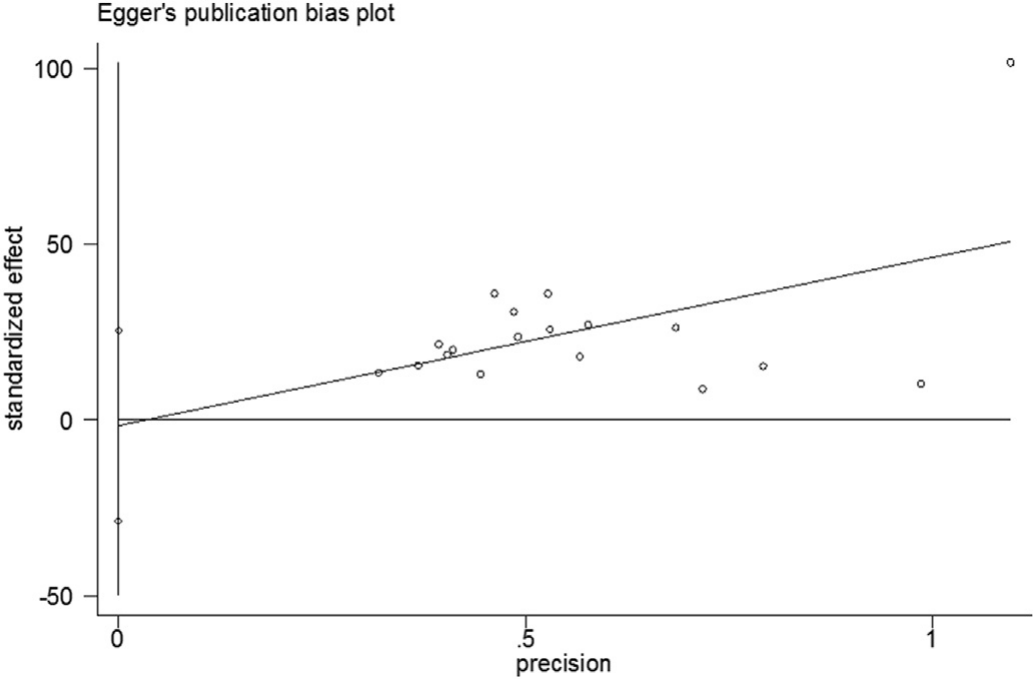
Displaying Egger's publication bias plot.

Visual inspection of the funnel plot and Egger's regression test were used to determine the presence of publication bias. Visual inspection of the funnel plot suggested asymmetrical distribution of included studies (Figure 3). The result of Egger's test (p = 0.898) showed that there was no possibility of publication bias (Figure 4).

### 2.12 Factors associated with postpartum modern contraception (PPMC) use

In this systematic review and meta-analysis, having prenatal family planning counseling, postnatal care utilization, spouse communication on family planning, and resumption of menses and sexual activity were the associated factors for the utilization of postpartum family planning (Table 3).

**Table 3:** Factors associated with PPMC utilization in Ethiopia, 2019.

Variables	AOR with 95% CI	I^2^ % & P- value	Model	Egger test
Prenatal family planning counselling	3.80 (2.70, 5.34)	0.0 & 0.682	Fixed	0.734
Postnatal care utilization	3.07 (1.39, 6.77)	91.1 & ≤0.001	Random	0.930
Spouse communication on family planning	1.86 (1.36, 2.54)	0 & 0.468	Fixed	0.065
Resumption of menses	4.20 (2.95, 5.99)	75.5 & ≤0.001	Random	0.634
Resumption of sexual activity	3.98 (2.34, 6.79)	62.6 & 0.030	Random	0.076

## 3 Discussion

This systematic review and meta-analysis were aimed at estimating the overall pooled prevalence of postpartum modern contraceptive (PPMC) use in Ethiopia.

Increased birth intervals and reduced unwanted pregnancy, as well as its implications, require postpartum family planning. If mothers begin using it as soon as feasible after delivery, it can reduce maternal and child mortality [44]. Therefore, the finding of this meta-analysis showed that the overall pooled prevalence of PPMC use in Ethiopia among postpartum women was 45.44% (95%CI: 31.47, 59.42).

In the individual studies, the prevalence of PPMC use among postpartum women varied from 10.30 % in a study conducted in Debat district, Amhara [41] to 92.80 % in Addis Ababa, Ethiopia [43].

This pooled prevalence is congruent with the recent national mini demographic health survey report from the general population which showed that the modern contraceptive use among married women in Ethiopia was 41% [45]. This could indicate that PPMC use is uncommon, given that postpartum family planning is one of the recommended public health interventions for reducing maternal and child morbidity and mortality [2, 46], and the first year after a woman gives birth is an important period for contraceptive uptake to avoid unintended pregnancy [4]. Postpartum women were more likely to use contraceptives than the general population. Despite the fact that the postpartum period is an optimum time to begin family planning, it is frequently disregarded. This could be because even if they are breastfeeding, postpartum women may not recognize they are at risk of pregnancy [47]. This finding was in line with previous studies conducted in low- and middle-income countries in which the overall pooled modern contraceptive prevalence rate (mCPR) during the postpartum period across all regions was 41.2% [48].

Furthermore, this finding is also almost similar to that of single studies conducted among postpartum women in Mexico (47%) [49], in Rwanda (50%) [50], and evidence from population surveys in Kenya and Zambia (45.9%) [51].

The prevalence noted in this study was higher than the postpartum contraceptive prevalence revealed by the Demographic and Health Survey (DHS) data-based study in Ethiopia (23%) [14]. This could be the fact that Demographic and Health surveys may include more postpartum women residing in rural areas with a home delivery history. As a result, they may not have the opportunity to obtain maternal health services.

Similarly, the finding of this meta-analysis was higher than that reported in a single study done in Uganda (28%) [52], and studies including different countries using their respective demographic health survey data (2008–2012 DHS data); Ethiopia (8%), Comoros (6.4%) and Burundi (5.8%) [53]. This might be due to the difference in the study period, socioeconomic status of the study participants, and also accessibility of the health services, and improvement in health service delivery. Ethiopian government attempts to strengthen maternal and child health morbidity and mortality reduction strategies, such as community and institutional-based reproductive health services and health education provided by health workers on a house-to-house basis, may also explain this finding, implying that introducing a women's development army and community health insurance [54] could have a positive effect on the rising rates.

However, these systematic review and meta-analysis estimates were found to be lower than those reported in individual studies conducted in Malawi (74.6%) [55], South Africa (89.0%) [56], and Kenya, Nairobi [57]. The possible explanation for this variation might be the presence of socioeconomic differences and sociocultural status variations, availability, and service accessibility.

Using the eggers regression test, we were unable to find publication bias in this meta-analysis. The lack of significant dispersion in the sample among the selected studies could explain the non-significant publication bias in the regression test. Evidence suggests that non-significant eggers' regression tests can be caused by a lack of substantial dispersion and a small sample size [58]. The other aim of this systematic review and meta-analysis was to identify the commonest associated factors with PPMC utilization in Ethiopia. Prenatal (antenatal) family planning counseling, postnatal care utilization, spouse communication on family planning, resumption of menses, and resumption of sexual activity were the most common factors that contributed to PPMC utilization in Ethiopia.

The result of this meta-analysis has found that family planning counseling during ANC contributed to modern contraceptive use during the postpartum period. Participants in the ANC who received family planning advice were more than three times as likely to utilize postpartum modern contraceptive methods than their counterparts. This result was in agreement with a systematic review and meta-analysis of postpartum contraceptive use among women in low- and middle-income countries [48]. Additionally, this finding was also similar to studies conducted in Mexico [49], the United States of America [59], North America [60], Malawi [55], Nigeria [61], and Bolivia, Egypt, and Thailand [62]. This could be since women who received family planning counseling would have a better understanding of the different types of family planning methods along with their merits and demerits, and increased awareness of birth spacing by the use of contraceptives after giving birth would enhance their decision-making skills towards postpartum family planning and motivate them to adopt contraceptives. Therefore, integrating FP services into the ANC may be a successful approach to pregnant women to establish an informal forum that will help them to discuss and share information, create a good attitude and strengthen intentions towards utilization of postpartum family planning. Evidence from studies that rely on DHS data in Kenya and Zambia [51] and studies from the Ethiopian Demographic and Health Surveys (EDHS) data [14] shows that ANC contributes to enhancing postpartum family planning utilization. Similarly, another systematic review also reported that integration of family planning into antenatal care services showed increased modern family planning use [63].

The utilization of postnatal care (PNC) services was found as a significant determinant of modern contraceptive uptake during the postpartum period. This systematic review and meta-analysis identified that women who had PNC service utilization were about three times more likely to use PPMC methods. Similarly, studies conducted in Mexico [49], and Kenya, and Zambia [51] found a similar result. This finding could be explained by the fact that women who received postnatal care are allowed to seek contraceptive advice and consider adoption.

Spouse communication on family planning was considered as an important driving force for practicing postpartum modern contraception in the present study. Mothers who had discussed contraception with their partners during the postpartum period were approximately two times more likely to utilize modern contraceptive methods than their counterparts. This relationship is also evidenced by other studies' findings conducted in Malawi [55], Nigeria [64], and Congo [65]. According to the EDHS 2011 report, women who have discussed FP with their husbands have a lower unmet need for FP than their counterparts [66]. This conclusion could be explained by the fact that interactions with their husbands can provide them with additional knowledge, support, and decisions about whether or not to use maternal health services, as the issue of family planning is not solely a concern of one partner, so this could increase their intention to use contraception after delivery. In this study, return of the menstrual cycle was revealed to be one of the most strongly associated factors to contraceptive use. Those who had their menses return after their last delivery were four times more likely to start using modern contraception on time than women who had amenorrhea after their last delivery. . A meta-analysis undertaken in poor and middle-income countries [48] supported this conclusion. This finding was also supported by reports from India [67], Malawi [55], Nairobi [68], and a Demography Health Survey-based analysis from 27 countries [5]. Furthermore, evidence reveals that women wait until their period has returned before using contraception, and they are unaware that they are at danger of becoming pregnant, even if they are amenorrheic [69, 70]. This could be related to moms' beliefs that fertility returns only when menses return, prompting them to adopt contraceptive techniques. Furthermore, women with amenorrhea would believe that they were less likely to become pregnant, considering that amenorrhea might prevent pregnancy regardless of the postpartum time. A woman may, however, ovulate before her first menstruation after childbirth, putting her at risk of pregnancy until she begins menstruation [13, 69].

Resumption of sexual activity was the other variable that demonstrated a statistically significant link with postpartum modern contraceptive use. When women resumed sexual activity, they were approximately four times more likely to utilize modern contraception after giving birth. Similar outcomes have been documented in Malawi [55], Egypt, Bolivia, and Thailand [62] study. This could be due to the fact that women who resume sexual activity fear becoming pregnant, encouraging them to seek out and use contraception.

## 4 Conclusion and recommendations

In Ethiopia, postnatal women used modern contraceptives at a low rate during the first year following delivery. Family planning counseling during ANC, PNC utilization, spouse communication on family planning, resumption of menses, and resumption of sexual activity were factors significantly associated with postpartum modern contraceptives’ uptake. As a result, integrating family planning services with pregnancy-postpartum care, encouraging couples to adopt tailored discussion approaches on family planning services, delivering effective public messages for couples on pregnancy risks before the menstruation returns, and conveying messages to couples about having unprotected sexual intercourse putting them at risk of becoming pregnant are all priorities.

### 4.1 Strengths and limitations

• The strength of this study is that it includes wide geographical areas and different eligible articles across the country, which increases the accuracy of the finding.
• The Preferred Reporting Items for Systematic Reviews and Meta-Analyses was used for result reporting.
• All the included studies in this systematic review and meta-analysis were cross-sectional studies.
• This systematic review and meta-analysis include articles published only in English.
• Contraceptive needs and use can change throughout the postpartum period, hence our analyses may not reflect these changes over time.

## Declarations

### Author contribution statement

Azimeraw Tesfu, Fentahun Beyene, Fikadu Sendeku, Kihinetu Wudineh and Getnet Azeze: Conceived and designed the experiments; Performed the experiments; Analyzed and interpreted the data; Contributed reagents, materials, analysis tools or data; Wrote the paper.

### Funding statement

This research did not receive any specific grant from funding agencies in the public, commercial, or not-for-profit sectors.

### Data availability statement

Data will be made available on request.

### Declaration of interests statement

The authors declare no conflict of interest.

### Additional information

No additional information is available for this paper.
